# Entrepreneurial Institutional Environment and Entrepreneurial Orientation: The Mediating Role of Entrepreneurial Passion

**DOI:** 10.3389/fpsyg.2022.840548

**Published:** 2022-04-15

**Authors:** Xue Zhou, Ling Zhang, Xiaoyun Su

**Affiliations:** Business School, Qingdao University, Qingdao, China

**Keywords:** entrepreneurial institutional environment, entrepreneurial orientation, entrepreneurial passion, cognitive appraisal theory of emotion, entrepreneurial enterprises

## Abstract

The entrepreneurial institutional environment is the external factor that entrepreneurial enterprises rely on for survival. Our interest is in how entrepreneurs cultivate entrepreneurial orientation in response to the highly uncertain entrepreneurial situation. Based on the cognitive appraisal theory of emotion, we analyzed the impact of the entrepreneurial institutional environment on entrepreneurial orientation through entrepreneurial passion. This study applied stepwise regression analyses to test the hypotheses on a sample of 197 entrepreneurs from the co-creation space in China. The output of the study showed that the entrepreneurial institutional environment had a positive effect on entrepreneurial orientation and that entrepreneurial passion played a mediating role between the entrepreneurial institutional environment and entrepreneurial orientation.

## Introduction

The entrepreneurial institutional environment is the external factor that entrepreneurial enterprises rely on for survival. A related study points out that the entrepreneurial institutional environment is the key to developing entrepreneurial enterprises. From the perspective of the relationship between the system and the organization, it is found that the entrepreneurial institutional environment has a significant impact on entrepreneurial activities. Manolova et al. (2008) and Stenholm et al. (2013) found that systematic differences in different regions significantly affect the entry rate of entrepreneurial activities, entrepreneurial types, and other entrepreneurial activities. However, this kind of research mainly discusses the mechanism of the impact of the institutional environment on entrepreneurial enterprises from the macro-environmental or corporate perspective, ignoring the micro-effects of the institutional environment on entrepreneurs.

An institutional theory emphasizes the embeddedness of organizations, pointing out that the growth trajectory of individuals and organizations will be affected by the social environment in which they are located. The cognitive appraisal theory of emotion points out that the influence of stimulus on individual emotions does not come from the stimulus itself but the individual’s cognitive evaluation of the stimulus (Smith and Ellsworth, 1987). Entrepreneurial institutional environment is a specific institutional environment concept for entrepreneurial activities, including three dimensions of regulation, norms, and cognition. It is a vital constraint for developing entrepreneurial activities (Manolova et al., 2008). At the helm of entrepreneurial enterprises, their emotions, especially entrepreneurial passion, which is the unique emotion of entrepreneurs, will inevitably change due to the stimulation of the entrepreneurial institutional environment (Cardon et al., 2013). At the same time, in discussing the relationship between emotion and motivation, the cognitive appraisal theory of emotion also explains people’s behavior. Arnold (1971) illustrates the relationship between emotion and motivation by constructing an “action sequence” model. The model theory assumes that humans are “doers” who are inherently motivated to act in response to environmental stimuli. This process of emotion-generated behavior can be described as “cognition-evaluation-emotion-generating-need-think-action” (Arnold, 1971). It can be seen that the cognitive appraisal theory of emotion not only focuses on how emotions are generated but also explains the influence and determinative role of emotions on individual behavior.

As a unique emotional manifestation of entrepreneurs, entrepreneurial passion is a typical fusion of entrepreneurial activities and emotional cognition (Cardon et al., 2013). Previous studies have suggested that the entrepreneurial passion experience may vary based on the entrepreneur’s different stages of enterprise development, or it may vary due to the entrepreneur’s diverse background and life experience, including the entrepreneur’s age, gender, education level, and life span of the enterprise—the number of companies established in the past, etc. (Cardon et al., 2013). Türk et al. (2020) found that pre-entrepreneurship experience (i.e., entrepreneurial role model experience and direct entrepreneurial experience) impacts the development of entrepreneurial passion (Türk et al., 2020). It should be noted that the discussion on the inducing factors of entrepreneurial passion is mainly from the perspective of entrepreneurs’ factors, and there is a lack of a debate on external stimulus. Murnieks et al. (2014) found that social factors can affect the generation of entrepreneurial passion (Murnieks et al., 2014). Still, it is with the Dualistic Model of Passion, which argues that individuals can develop feelings of passion either harmoniously (autonomously) or obsessively (compulsively) (Vallerand, 2015). Previous studies have found that the impact of entrepreneurial passion is analyzed from the aspects of entrepreneurial intention, entrepreneurial behavior, venture growth, etc. On the one hand, maintaining entrepreneurial passion is beneficial for entrepreneurs, as it enables them to learn from their entrepreneurial experience, form correct risk perception, and reasonably prevent risks (Cardon and Kirk, 2015). On the other hand, entrepreneurial enterprises can drive entrepreneurs to persist and adopt innovative practices of entrepreneurial behavior to meet various entrepreneurial challenges and achieve entrepreneurial enterprise growth (Baum and Locke, 2004; Li et al., 2020). In this study, we use the cognitive appraisal theory of emotion as a lens to integrate considerations of the entrepreneurial institutional environment into the development of entrepreneurial passion. In addition, based on the logical framework of “stimulus-organism-response,” we analyze the impact of the entrepreneurial institutional environment on entrepreneurial orientation through entrepreneurial passion.

The contribution of this study lies in the following aspects. First, from the perspective of the cognitive appraisal theory of emotion, we reveal the positive effect of the entrepreneurial institutional environment as an external stimulus on entrepreneurs’ unique emotions, namely, entrepreneurial passion. This enriches the study of antecedents of entrepreneurial passion to a certain extent and further highlights the importance of the entrepreneurial institutional environment. Second, the research on the antecedents of entrepreneurial orientation has been further enriched. This study explores how the entrepreneurial institutional environment affects entrepreneurial-oriented strategies. In particular, we integrate the study of entrepreneurial passion with the theoretical framework developed by Cardon et al. (2009, 2013) to show that the paths to intense positive feelings and identity centrality are different. Third, based on the entrepreneur’s cognitive evaluation of the emotion process, this study constructs the stimulus process of the external environment to individual behavior as a process of emotional response. We establish the theoretical logic of “stimulus-organism-response,” verify it with empirical data, and ultimately realize the fitting of entrepreneurial practice and cognitive appraisal theory of emotion, which expands the research on the cognitive appraisal theory of emotion in the field of entrepreneurship.

## Theoretical Review and Research Hypotheses

### Entrepreneurial Institutional Environment

The institutional theory believes that socialization activities, such as entrepreneurship, depend on the specific institutional context (Peng, 2003). The entrepreneurial institutional environment is a straightforward concept for entrepreneurial activities, including three dimensions of regulation, norms, and cognition. It is a vital constraint for developing entrepreneurial activities (Manolova et al., 2008). The regulatory dimension measures the government’s support for entrepreneurial enterprises in policies and systems. The normative dimension measures the recognition and admiration of entrepreneurial activities at the social level. The cognitive size measures people’s entrepreneurial-related information, knowledge, skills, etc. The degree of mastery (Manolova et al., 2008). Some scholars have researched the influence of the external institutional environment on entrepreneurial activities, which more uniformly reflects the positive effects of the external institutional environment on entrepreneurial practice. Studies have shown that a sound regulatory environment, such as government support policies, tax relief, etc., can effectively support the maturity of startups (Stenholm et al., 2013; Brush et al., 2019). Social norms and systems with a high degree of entrepreneurial recognition, such as a social atmosphere that advocates entrepreneurship, are conducive to promoting the growth of entrepreneurial enterprises (De Clercq et al., 2010). A high-level cognitive environment means rich entrepreneurial experience and knowledge. Estrin and Mickiewicz (2012) encourage forming strong support for entrepreneurial activities. Entrepreneurs receive higher returns from an entrepreneurial strategic posture in countries where institutions (legal and financial systems), entrepreneurial education, and cultural support for entrepreneurship are more developed (Wales et al., 2021). However, the study mentioned earlier mainly discusses the institutional environment’s direct effect or moderating effect at the organizational level and lacks a discussion on the micro-mechanism of entrepreneurs’ cognitive status.

### Entrepreneurial Passion

As a unique emotional manifestation of entrepreneurs, entrepreneurial passion is a typical fusion of entrepreneurial activities and emotional cognition (Cardon et al., 2013). Entrepreneurial passion is the source of motivation for entrepreneurial activities. At present, scholars mainly understand the connotation of entrepreneurial passion from the perspective of entrepreneur traits, emotions, and motivation (Newman et al., 2021). The trait perspective emphasizes the innateness of entrepreneurial passion as an innate endowment of entrepreneurs (Baum et al., 2001). The philosophy emphasizes the emotional experience of entrepreneurial passion and regards it as the emotional support of entrepreneurs’ aggressive and unremitting efforts (Cardon et al., 2009, 2017). The motivational perspective emphasizes the stimulating effect of entrepreneurial passion on entrepreneurial behavior, which is regarded as an inducing factor for entrepreneurs to move forward firmly (Laaksonen et al., 2011; Cardon and Kirk, 2015). According to the classification of entrepreneurial passion in Cardon’s study, entrepreneurial passion includes two dimensions: intense positive feelings and identity centrality (Cardon et al., 2013). Intense positive feelings are the positive and lasting emotions that entrepreneurs consciously maintain in entrepreneurial activities, and identity centrality includes social identity and self-identification. Social identity comes from others’ recognition of the entrepreneur’s identity, and self-identity comes from the entrepreneur’s conversion of roles accepted (Cardon et al., 2013).

### Entrepreneurial Orientation

Entrepreneurial orientation is the continuous strategic behavior of managers pursuing innovation and development motivation (Covin and Slevin, 1989; Srivastava and Lee, 2005; Stewart et al., 2021), which reflects the decision-making tendency to develop new opportunities driven by management cognition, and is mainly reflected in the entrepreneurial independence in a complex and dynamic market environment. Covin and Wales (2012) believes that entrepreneurial orientation is embodied in innovativeness, risk-taking, and proactiveness (Covin and Wales, 2012). Innovativeness refers to the company’s tolerance for new ideas, experiments, and creativity as a source of competitive advantage. Risk-taking refers to the willingness of an enterprise to make a large number of risk resource commitments. Proactiveness reflects the company’s tendency to actively compete with competitors (Patel et al., 2015; Bouncken et al., 2016).

### Entrepreneurial Institutional Environment and Entrepreneurial Passion

According to the cognitive appraisal theory of emotion, emotion is an individual’s adaptive response to specific events in the external environment. Moreover, emotions arise from the individuals’ subjective mental evaluations of the external stimulus events. Once individuals perceive the external environment, they will evaluate this external stimulus information in a meaningful direction (Smith and Ellsworth, 1987; Moors et al., 2013). As an external stimulus, the entrepreneurial institutional environment impacts entrepreneurs’ unique emotions and entrepreneurial passion.

The entrepreneurial institutional environment affects the intense positive feelings. First, the social level of respect for entrepreneurial activities can make entrepreneurs’ ideas and opinions more recognized. This kind of support from the environment can bring positive feedback to entrepreneurs, enhance their self-confidence, and thus strengthen their active and lasting entrepreneurial activities (Cardon et al., 2013). However, in a social context that does not encourage entrepreneurship, entrepreneurs feel the prejudice against entrepreneurship in society, undermining individual confidence in successful entrepreneurship (Anokhin and Schulze, 2009). Second, identifying opportunities is endogenous in the interaction between entrepreneurs and the environment (Holcombe, 2003). The government’s policy and institutional support for entrepreneurial enterprises provide entrepreneurs with a basis for continuous experimentation and exploration. Even if they encounter failure, they can still obtain resources to develop new entrepreneurial opportunities, which will not make entrepreneurs lose their passion in the loss, and help entrepreneurs maintain a lasting enthusiasm for entrepreneurial activities. In addition, generally, when entrepreneurs have rich entrepreneurial experience, knowledge, and skills, they will be more inclined to utilize their knowledge and skills to start and run enterprises, and their entrepreneurial attitudes will be more willing to think independently and take risks (Sambharya and Musteen, 2014). Entrepreneurs can amplify their positive emotions for entrepreneurship (Newman et al., 2021). Accordingly, we hypothesize,

H1: Entrepreneurial institutional environment is positively associated with intense positive feelings.

The identity centrality in entrepreneurial passion can establish entrepreneurs’ belief to maintain their identity. According to the cognitive appraisal theory of emotion, the emotional response of entrepreneurs is determined by their cognitive evaluation of specific stimulus events (Arnold, 1971). Once the stimulus event is perceived, the individual will automatically evaluate “it is good or bad,” producing a positive or negative emotional response related to the stimulus event (Moors et al., 2013).

The entrepreneurial institutional environment affects identity centrality. First, the government’s policy and institutional support for entrepreneurial activities can provide substantial assistance for them to solve their difficulties and overcome obstacles, ease the pressure that entrepreneurs bear in the entrepreneurial process, and make entrepreneurs feel affirmed, thereby enhancing their identity centrality (Klyver et al., 2018). Second, the recognition and admiration of entrepreneurial activities at the social level may enable entrepreneurs to make more positive evaluations of the activities they are engaged in, think that what they do is valuable, and be more optimistic about their attitude toward entrepreneurial activities. This will shape the entrepreneur’s sense of identity and belonging to entrepreneurial activities (Klyver et al., 2018). Third, people’s grasp of entrepreneurial-related information, knowledge, and skills helps them understand entrepreneurial behavior, clarify the social value of entrepreneurial activities, and recognize the social role of entrepreneurs. Barba-Sánchez et al. (2022) found that the knowledge and values linked to sustainability (i.e., environmental awareness) motivate individual entrepreneurs to engage in environmentally friendly practices. When entrepreneurs perceive support and understanding from society, it can prompt them to form a positive evaluation of their identity. Accordingly, we hypothesize,

H2: Entrepreneurial institutional environment is positively associated with identity centrality.

### The Mediating Role of Entrepreneurial Passion

According to the cognitive appraisal theory of emotion, when people perceive an external stimulus, they evaluate the stimulus and then produce emotional responses. Emotional responses will trigger the need for action. These selected needs constitute motivation and drive people to take corresponding measures (Gasper and Bramesfeld, 2006). Entrepreneurial orientation is the continuous strategic behavior of managers pursuing innovation and development motivation (Srivastava and Lee, 2005). Entrepreneurial passion is a crucial factor that drives the individual behavior of entrepreneurs (Cardon et al., 2005). As a positive emotion peculiar to entrepreneurs, entrepreneurial passion can drive entrepreneurs to actively and optimistically carry out entrepreneurial activities (Stone and Glass, 1986; DeSteno et al., 2000). Cardon et al. (2009) found that entrepreneurship is essential in entrepreneurial activities because entrepreneurial passion is critical to persevere when encountering difficulties.

Entrepreneurial passion includes two parts: intense positive feelings and identity centrality. Intense positive feelings enable the entrepreneurs to persevere in entrepreneurial activities, identify information in the environment, solve creative problems, and pursue creative routes of action (Cardon et al., 2009, 2013; Liu et al., 2011). Entrepreneurs can face setbacks and failures in entrepreneurship with an optimistic attitude. Entrepreneurial passion means that entrepreneurs dare to overcome difficulties and can continue to think creatively during the entrepreneurial process (Schindehutte et al., 2006). Even in the face of setbacks or failures, they can still flexibly use information processing and resource patching strategies to face the risks and threats of the survival and development of entrepreneurial enterprises and spare no effort to promote entrepreneurial activities, which will help enhance the entrepreneurial orientation of innovativeness, risk-taking, and proactiveness (Murnieks et al., 2014). For example, in the Internet age, where market competition follows the rule of “fast fish eat slow fish,” positive entrepreneurial sentiment can prompt entrepreneurs to keep trial and error and iterate quickly to gain a competitive advantage (Murnieks et al., 2016).

Accordingly, we hypothesize,

H3: Intense positive feelings are positively related to entrepreneurial orientation.

After pointing out that the environment can trigger individual emotional responses, the cognitive appraisal theory of emotion further argues that emotions can lead to specific behaviors; that is, entrepreneurial orientation may be an emotional response to identity centrality (Arnold, 1971). The stronger the sense of identity centrality, the more able to persist in participating in activities related to that identity (Houser-Marko and Sheldon, 2006). On the one hand, entrepreneurs’ self-identification can effectively urge them to persevere in their role changes and strengthen entrepreneurs’ belief in achieving entrepreneurial goals. As Barba-Sánchez and Atienza-Sahuquillo (2017) note, motivation plays an important role in encouraging entrepreneurship. Motivated by identity centrality, entrepreneurs actively practice entrepreneurial orientation. On the other hand, the transformation of the role of entrepreneurs is consistent with residents’ perceptions of entrepreneurial identity. This is conducive to startups gaining legitimacy, building advantages in information integration and resource acquisition, and helping startups identify shortcomings and adjust strategies promptly on time (Hoang and Gimeno, 2010). Accordingly, we hypothesize,

H4: Identity centrality is positively related to entrepreneurial orientation.

Comprehensively considering Hypotheses 1–4, we put forward the mediation hypothesis of intense positive feelings and identity centrality.

H5: Intense positive feelings play a mediating role between entrepreneurial institutional environment and entrepreneurial orientation.
H6: Identity centrality plays a mediating role between entrepreneurial institutional environment and entrepreneurial orientation.

**FIGURE 1:**
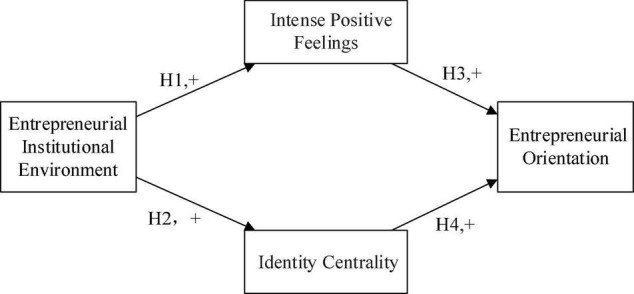
Conceptual model.

The theoretical model of this study is shown in Figure 1.

## Research Design

### Sample Selection and Data Collection

According to Cardon and Kirk (2015), this study focuses on the founders or co-founders of new ventures. It uses a questionnaire survey method to collect data and then tests the conceptual model and hypotheses to pursue the generalizability of the findings. The data source for this study comprised of the data collected particularly from June to October 2019 in the eastern entrepreneurial region of Mainland China, which enjoyed relatively high financial support, with the advantages of a sufficient supply of raw materials and many engineering universities nearby. Referring to the definition of new ventures by McDougall and Robinson (1990) and Zahra (1993), a new venture is an enterprise that is established for 8 years or less (McDougall and Robinson, 1990; Zahra, 1993). The entrepreneurs were asked to complete the questionnaire. We mainly looked for entrepreneurs in the co-creation space. First, we took a manuscript questionnaire and some small souvenirs to visit the entrepreneurs in the co-creation space one by one. Second, we will contact the management department of the co-creation space. They have a WeChat group that includes all the entrepreneurs in the area. Upon our request, they will send the questionnaire link to the group to retrieve the electronic version of the questionnaire.

We used three methods to minimize general method deviation. The first was by implementing anonymous filling methods to reduce the person’s responsibilities while filling the questionnaire. The second was to avoid the questionnaire being filled out multiple times by the same person (filling in online and offline questionnaires at once). Third, we required the new ventures to collect only one sample to avoid comparing the subjects of the same company. We distributed 256 questionnaires. After eliminating over five unanswered questionnaires, 197 valid questionnaires were left. The correct response rate was 76.9%. Among the 197 respondents, 68.0% were men and 32.0% were women. About 0.5% were 20 years or younger, 48.7% were between 20 and 30 years, 47.7% were between 30 and 40 years, and 3.0% were 40 years or older. Most respondents held a bachelor’s degree (71.1%), and 18.2% had a master’s degree or higher. The respondents were first-time entrepreneurs (57.4%), second-time entrepreneurs (31.5%), and third-time or more entrepreneurs (11.1%). All new ventures had operated below 8 years, and most of them had worked for 3–5 years (51.1%). Table 1 lists the characteristics of the respondents.

**TABLE 1:** Characteristics of the respondents (*n* = 197).

	Value	Number of response	Percentage (%)
Gender	Male	134	68.0
	Female	63	32.0
	Total	197	
Age	20 or younger	1	0.5
	20–30	96	48.7
	30–40	94	47.7
	40 or older	6	3.0
	Total	197	
Educational background	High school or below	6	3.0
	Vocutuinal school	15	7.6
	University	140	71.1
	Master’s/Ph.D.	36	18.2
	Total	197	
Industry experience	Five years and below	110	55.8
	5–10 years	57	29.0
	Ten years and more	30	15.2
	Total	197	
Previous entrepreneurial experience	1	113	57.4
	2	62	31.5
	Three and more	22	11.1
	Total	197	
Firm age	Under 1	22	11.2
	1–2	44	22.3
	3–5	101	51.1
	6–8	30	15.3
	Total	197	
Firm size	20 and below	71	36.1
	20–50	63	32.0
	50–100	34	17.3
	100–200	26	13.2
	200 and more	3	1.5
	Total	197	

### Variable Measurement

For the adopted foreign scales, some authors first translate the survey items, if initially in English, into Chinese and use back-translation to test the accuracy (Craig and Douglas, 2006). We invite two Ph.D. candidates who major in psychology and management with the experience of studying in English-speaking countries for more than 2 years to translate them from English to Chinese. We review the original and back-translated versions to ensure they are equivalent. The questionnaire mainly includes background information of entrepreneurs or new ventures and measurement of variables. The background information of entrepreneurs or new experiences, such as age, education background, and firm size, is measured using the form of selection or filling in the blanks. The questionnaire measurement of variables, including entrepreneurial institutional environment, intense positive feelings, identity centrality, and entrepreneurial orientation, in this study utilizes a seven-point Likert scale with one indicating complete disagreement and seven indicating full compliance.

Entrepreneurial institutional environment. Based on the study of Stenholm et al. (2013), the scale contains nine items, such as “ease of starting a business,” “social status of entrepreneurs,” etc. The internal consistency of Cronbach’s alpha coefficient was 0.712.

Entrepreneurial passion. Based on the learnings from Cardon et al.’s (2013) study, we used a scale that contained 13 items. With 10 items measuring intense positive feelings, such as “it is exciting to figure out new ways to solve unmet market needs that can be commercialized,” “searching for new ideas for products/services to offer is enjoyable to me,” “I am motivated to figure out how to make existing products/services better,” etc. The internal consistency of Cronbach’s alpha coefficient was 0.775. With three items measuring identity centrality, such as “inventing new solutions to problems is an important part of who I am,” “being the founder of a business is an important part of who I am,” and “nurturing and growing companies is an important part of who I am.” The internal consistency of Cronbach’s alpha coefficient was 0.616. For the whole scale, the inner surface of Cronbach’s alpha coefficient was 0.816.

Entrepreneurial orientation. According to Covin and Slevin (1989), we use a nine-item scale, such as “after the establishment of a new venture, many new products or new services have been developed,” “The company will first initiate a competitive action, and then the competitor will be forced to respond,” “the corporate management team prefers high-risk projects with high returns,” etc. The internal consistency of Cronbach’s alpha coefficient was 0.745.

Control Variables. With reference to the existing research methods, gender, age, educational background, industry experience, previous entrepreneurial experience, firm age, and firm size are used as control variables.

### Common Method Bias

We used Harman’s single-factor test to perform an unrotated factor analysis on all the collected questionnaire data to test the common method bias. The variance explained by the first principal component was 25.09%, which does not constitute half of the variance explained by the total variables (60.43%). Therefore, the common method bias of the sample data was within an acceptable range.

## Empirical Analyses and Results

### Convergent Validity

Convergent validity was a measure of the model fit. The average variance extracted (AVE) showed the degree of correlation between the construct and its indices, with a greater fit achieved with a stronger correlation. Any composite-reliability (CR) rating higher than 0.7 (Hair et al., 2017) suggests that the construct was internally acceptable. In this study, the AVE of all the variables was higher than 0.5, and the CR of all the variables was higher than 0.7 (Table 2).

**TABLE 2:** Indicators of measurement.

Variable	Items	Factor loading	Average variance extracted (AVE)	Composite reliability (CR)	Cranach’s alpha
Entrepreneurial institutional environment	EIE1	0.840	0.538	0.912	0.712
	EIE2	0.762			
	EIE3	0.872			
	EIE4	0.713			
	EIE5	0.663			
	EIE6	0.705			
	EIE7	0.664			
	EIE8	0.667			
	EIE9	0.682			
Entrepreneurial passion	IPF1	0.848	0.544	0.936	0.816
	IPF2	0.866			
	IPF3	0.429			
	IPF4	0.803			
	IPF5	0.617			
	IPF6	0.819			
	IPF7	0.918			
	IPF8	0.926			
	IPF9	0.474			
	IPF10	0.614			
	IC1	0.779			
	IC2	0.688			
	IC3	0.594			
Entrepreneurial orientation	EO1	0.824	0.626	0.936	0.745
	EO2	0.822			
	EO3	0.834			
	EO4	0.581			
	EO5	0.753			
	EO6	0.800			
	EO7	0.927			
	EO8	0.577			
	EO9	0.925			

### Discriminant Validity

Discriminant validity is the extent to which a construct is truly distinct from other constructs by empirical standards (Hair et al., 2017). We conducted a series of confirmatory factor analyses to test the discriminant validity of the variables involved in this study. Following the suggestion of Hau and Marsh (2004), we first examined the baseline model (the four-factor model) that included four key variables: entrepreneurial institutional environment, intense positive feelings, identity centrality, and entrepreneurial orientation (Hau and Marsh, 2004). The four-factor model indices showed that the data fit well (χ^2^ = 525.31, RMSEA = 0.04, CFI = 0.92, TLI = 0.91, and SRMR = 0.06) and factor loadings were significant. To confirm the measurement model, the baseline model was contrasted with alternative CFA models. The alternative CFA models are presented in Table 3, and it can be seen that the four-factor model fitted the data considerably better than any of the alternative CFA models (Cheung and Rensvold, 2002). Hence, the discriminant validity of the four variables was confirmed.

**TABLE 3:** Confirmatory factor analysis results.

Models	χ^2^	df	χ^2^/df	RMSEA	SRMR	CFI	TLI
Four factors	525.31	399	1.32	0.04	0.06	0.92	0.91
Three factors a	535.63	402	1.33	0.04	0.06	0.90	0.91
Three factors b	590.78	404	1.46	0.05	0.06	0.88	0.86
Two factors c	592.00	406	1.46	0.05	0.06	0.88	0.86
One factor d	630.36	407	1.55	0.05	0.07	0.86	0.84

*(a) Entrepreneurial institutional environment + Identity centrality, Intense positive feelings, Entrepreneurial orientation; (b) Entrepreneurial institutional environment + Intense positive feelings, Identity centrality, Entrepreneurial orientation; (c) Entrepreneurial institutional environment + Identity centrality + Intense positive feelings, Entrepreneurial orientation; (d) Entrepreneurial institutional environment + Identity centrality + Intense positive feelings + Entrepreneurial orientation.*

### Descriptive Statistics

Table 4 presents the mean values, standard deviations, and correlations of all the variables. As given in Table 4, entrepreneurial institutional environment was positively correlated with intense positive feelings (*r* = 0.55, *p* < 0.01) and identity centrality (*r* = 0.51, *p* < 0.01), and intense positive feelings (*r* = 0.57, *p* < 0.01) and identity centrality (*r* = 0.46, *p* < 0.01) were positively correlated with entrepreneurial orientation. Moreover, all the square roots of the average variance extracted (AVE) of the constructs were higher than the correlation coefficients, suggesting the discriminant validity is confirmed (Fornell and Larcker, 1981).

**TABLE 4:** Descriptive statistical analysis.

	1	2	3	4	5	6	7	8	9	10	11
1. Gender	N/A										
2. Age	0.10	N/A									
3. Educational background	0.09	0.12	N/A								
4. Industry experience	0.01	0.58**	0.12	N/A							
5. Previous entrepreneurial experience	0.04	0.08	−0.06	0.11	N/A						
6. Firm age	0.01	0.20**	0.05	0.28**	0.22**	N/A					
7. Firm size	0.19**	0.14	0.16**	0.22**	0.28**	0.41**	N/A				
8. Entrepreneurial institutional environment	0.00	0.06	−0.03	0.19**	0.10	0.03	0.13	N/A			
9. Intense positive feelings	0.01	0.12	0.03	0.22**	0.01	−0.09	0.05	0.55**	N/A		
10. Identity centrality	−0.02	0.11	−0.00	0.14	−0.01	−0.15*	−0.05	0.51**	0.59**	N/A	
11. Entrepreneurial orientation	0.06	0.13	−0.11	0.12	−0.01	0.07	0.17*	0.63**	0.57**	0.46**	N/A
Mean	1.32	2.53	4.08	5.87	1.56	3.58	3.51	4.94	5.34	5.14	4.92
S.D.	0.47	0.57	0.72	3.57	0.77	1.88	1.66	0.75	0.80	1.03	0.81

N = 197. *Significantly correlated at the 0.05 level (bilateral). **Significantly correlated at the 0.01 level (bilateral).

### Model Design

Combined with theoretical deduction and research hypothesis, the regression model is established as follows:


(1) $$\begin{array}{c} IPF=\alpha_{1}+\alpha_{2}EIE\\ \;+\alpha_{i}Control_{gender,age,edu,ind,pre,firmage,firmsize}+\varepsilon \end{array}$$

(2) EO=α1′+α3IPF+αi′Controlgender,age,edu,ind,pre,firmage,firmsize+ε

(3) EO=α1″+α2′EIE+α3′IPF+αi″Controlgender,age,edu,ind,pre,firmage,firmsize+ε

(4) $$\begin{array}{c} IC=\beta_{1}+\beta_{2}EIE\\ \;+\beta_{i}Control_{gender,age,edu,ind,pre,firmage,firmsize}+\varepsilon \end{array}$$

(5) EO=β1′+β3IPF+βi′Controlgender,age,edu,ind,pre,firmage,firmsize+ε

(6) EO=β1″+β2′EIE+β3′IPF+βi″Controlgender,age,edu,ind,pre,firmage,firmsize+ε

### Hypothesis Testing

Stepwise regression analyses were conducted to test the hypotheses. In direct effects, we test the impact of the entrepreneurial institutional environment on intense positive feelings and identity centrality (Table 5).

**TABLE 5:** Regression analysis of direct effects.

Variables	Model 1	Model 2	Model 3	Model 4
		
	Outcome variable: intense positive feelings		Outcome variable: identity centrality	
Gender	−0.00 (−0.06)	0.00 (0.03)	−0.03 (−0.39)	−0.02 (−0.36)
Age	0.01 (0.05)	0.04 (0.52)	0.07 (0.76)	0.10 (1.31)
Educational background	−0.00 (−0.04)	0.03 (0.50)	−0.01 (−0.19)	0.02 (0.29)
Industry experience	0.25** (2.87)	0.14 (1.79)	0.16 (1.81)	0.05 (0.62)
Previous entrepreneurial experience	0.00 (0.03)	−0.03 (−0.50)	0.02 (0.27)	−0.01 (−0.19)
Firm age	−0.20** (−2.46)	−0.16** (−2.30)	−0.22 (−2.73)	−0.18** (−2.60)
Firm size	0.07 (0.91)	0.01 (0.19)	0.00 (0.01)	−0.06 (−0.82)
Entrepreneurial institutional environment		0.53*** (8.70)		0.51*** (8.10)
Intense positive feelings				
Identity centrality				
VIF maximum	1.60	1.65	1.60	1.65
*R* square	0.08	0.34	0.06	0.31
Δ *R* square	0.08	0.26	0.06	0.25
F	2.25**	12.20***	1.85*	10.36***

N = 197. ***, **, and * indicate p < 0.001, p < 0.01, and p < 0.05, respectively.

In H1 and H2, we posit that entrepreneurial institutional environment is positively related to intense positive feelings (H1) and identity centrality (H2). As given in Table 5, the influence of the coefficient of entrepreneurial institutional environment on intense positive feelings (β = 0.53, *t* = 8.70, *p* < 0.001) and identity centrality (β = 0.51, t = 8.10, *p* < 0.001) were all significantly positive. Therefore, H1 and H2 are supported. Specifically, when the entrepreneurial institutional environment supports entrepreneurship, the entrepreneurs have more intense positive feelings. Also, when the entrepreneurial institutional environment supports entrepreneurship, the entrepreneurs feel more identity centrality.

As presented in Table 6, we test the direct and mediation effects of intense positive feelings and identity centrality on entrepreneurial orientation. For immediate impact, in H3 and H4, we propose that intense positive feelings (H3) and identity centrality (H4) are positively related to entrepreneurial orientation. The results summarized in Table 6 show that both intense positive feelings (β = 0.59, *t* = 9.94, *p* < 0.001) and identity centrality (β = 0.49, *t* = 7.67, *p* < 0.001) show a positive effect on entrepreneurial orientation. Therefore, H3 and H4 are supported.

**TABLE 6:** Regression analysis of mediation effects.

Variables	Model 5	Model 6	Model 7	Model 8	Model 9
	
	Outcome variable: entrepreneurial orientation				
Gender	0.03 (0.43)	0.03 (0.57)	0.04 (0.74)	0.05 (0.71)	0.04 (0.81)
Age	0.10 (1.13)	0.10 (1.35)	0.13* (2.04)	0.07 (0.86)	0.12 (1.82)
Educational background	−0.17* (−2.38)	−0.17** (−2.90)	−0.14* (−2.76)	−0.17* (−2.62)	−0.14* (−2.52)
Industry experience	0.06 (0.65)	−0.09 (−1.25)	−0.13* (−2.04)	−0.02 (−0.27)	−0.10 (−1.44)
Previous entrepreneurial experience	−0.08 (−1.12)	−0.08 (−1.40)	−0.11* (−2.16)	−0.09 (−1.43)	−0.12* (−2.21)
Firm age	−0.02 (−0.25)	0.10 (1.44)	0.08 (1.41)	0.09 (1.21)	0.07 (1.11)
Firm size	−0.20* (−2.40)	0.15 (2.29)	0.12 (2.00)	0.20** (2.73)	0.13 (2.19)
Entrepreneurial institutional environment			0.47*** (7.72)		0.54*** (8.67)
Intense positive feelings		0.59*** (9.94)	0.34*** (5.48)		
Identity centrality				0.49*** (7.67)	0.21** (3.40)
VIF Maximum	1.60	1.67	1.68	1.63	1.65
*R* Square	0.07	0.39	0.54	0.29	0.50
Δ *R* Square	0.07	0.32	0.15	0.22	0.21
F	2.14*	15.18***	24.32***	9.80***	20.49***

N = 197. ***, **, and * indicate p < 0.001, p < 0.01, and p < 0.05, respectively.

From the perspective of the mediation effect, we assume that intense positive feelings (H5) and identity centrality (H6) play a mediating role between entrepreneurial institutional environment and entrepreneurial orientation. As shown in Table 6, the influence of the coefficient of entrepreneurial institutional environment on entrepreneurial orientation through intense positive feelings (β = 0.34, *t* = 5.48, *p* < 0.001) was significantly positive. The influence of the coefficient of entrepreneurial institutional environment on entrepreneurial orientation through identity centrality (β = 0.21, *t* = 3.40, *p* < 0.001) was significantly positive. Therefore, H5 and H6 are supported.

## Conclusion and Discussion

### Main Research Conclusion

The entrepreneurial institutional environment is the external factor that entrepreneurial firms rely on for survival. Entrepreneurial firms are a vital force supporting the country’s economic development. How entrepreneurs cultivate entrepreneurial orientation in response to the highly uncertain entrepreneurial situation has become a common concern. In this study, we use the cognitive appraisal theory of emotion as a lens to integrate considerations of the entrepreneurial institutional environment into the development of entrepreneurial passion. Based on the logical framework of “stimulus-organism-response,” taking founders or co-founders of new ventures as the study subjects, we analyze the impact of entrepreneurial institutional environment on entrepreneurial orientation through entrepreneurial passion. The main conclusions obtained are as follows.

First, the entrepreneurial institutional environment positively affects entrepreneurial passion, which is the unique emotion of entrepreneurs. This relationship can be explained from the perspective of cognitive appraisal of emotion. The social level of respect for entrepreneurial activities, government support in policies, and the mastery of entrepreneurial-related abilities can help entrepreneurs make positive emotional evaluations and decisions, maintain a lasting enthusiasm for entrepreneurial activities, and enhance their sense of identity. This finding echoes the view of Cardon et al. (2013) that environmental support gives entrepreneurs positive feedback, enhances entrepreneurs’ self-confidence, and thus strengthens their motivation to carry out entrepreneurial activities. From the perspective of cognitive appraisal of emotion, this conclusion shows that the entrepreneurial institutional environment, as a critical constraining situation for the development of entrepreneurial activities, affects the micro-mechanism of entrepreneurs’ cognitive level.

Second, the entrepreneurial institutional environment positively affects entrepreneurial orientation, and entrepreneurial passion plays a mediating role between the entrepreneurial institutional environment and entrepreneurial orientation. The study results show that entrepreneurial passion is beneficial for entrepreneurs, as it allows them to learn from entrepreneurial experience and enhances the entrepreneurial orientation of innovativeness, risk-taking, and proactiveness. This finding echoes the conclusion that Murnieks et al. (2014) pointed out in a study that entrepreneurial passion makes entrepreneurs face setbacks or failures and promotes their entrepreneurial-oriented cultivation. On the one hand, entrepreneurial passion, as a positive emotion, enables entrepreneurs to face setbacks and failures in entrepreneurship with an optimistic attitude, identify information in entrepreneurial activities, think creatively, solve problems, and cultivate entrepreneurial orientation. On the other hand, entrepreneurs with a stronger sense of identity will be able to participate in entrepreneurial activities and gain legal status for the enterprise, thereby supporting the realization of innovative, adventurous, and transformative entrepreneurial orientation.

### Theoretical Contribution

The theoretical contributions of this research are reflected in the following three aspects.

First, previous studies regarding the antecedents of entrepreneurial passion are mainly from the perspective of entrepreneurs’ factor, including the entrepreneur’s age, gender, education level, and life span of the enterprise—the number of companies established in the past, etc. (Cardon et al., 2013). There is a lack of a debate on external stimulus. In this study, we use the cognitive appraisal theory of emotion as a lens to integrate considerations of the entrepreneurial institutional environment into the development of entrepreneurial passion. This enriches the study on antecedents of entrepreneurial passion to a certain extent and further highlights the importance of the entrepreneurial institutional environment.

Second, in response to the call of Bouncken et al. (2016), the research on the antecedents of entrepreneurial orientation has been further enriched. In the past, discussions on the antecedents of entrepreneurial orientation mainly focused on external factors (e.g., hostility, complexity, dynamics, and richness of the environment) (Luo et al., 2005) or internal factors (e.g., the single perspective of the company’s technical capabilities, organizational resources, and leadership styles) (Ireland et al., 2001). Based on the cognitive appraisal theory of emotion, this study explores how the entrepreneurial institutional environment affects entrepreneurial-oriented strategies. In particular, we integrate the study of entrepreneurial passion into the theoretical framework developed by Cardon et al. (2009, 2013) to show that the paths to intense positive feelings and identity centrality are different.

Third, based on the entrepreneur’s cognitive evaluation of the emotion process, this study constructs the stimulus process of the external environment to individual behavior as a process of emotional response. We establish the theoretical logic of “stimulus-organism-response,” verify the mediating role of entrepreneurial passion between the entrepreneurial institutional environment and entrepreneurial orientation with empirical data, and ultimately realize the fitting of entrepreneurial practice and cognitive appraisal theory of emotion. The verification of emotional mechanisms provides a new theoretical perspective for studying the entrepreneurial institutional environment.

### Management Enlightenment

Based on the research conclusions of this study, the following management enlightenment can be obtained.

First, because the entrepreneurial institutional environment positively affects entrepreneurial passion, the government can provide political and institutional support related to entrepreneurial activities to help entrepreneurs solve difficulties, overcome obstacles, ease the pressure, and make entrepreneurs feel affirmed, enhancing their interest in entrepreneurship. That is, not to encourage non-market-oriented competitive behavior but to consider the micro-effects of the institutional environment on entrepreneurs. The government should consider entrepreneurial enterprises’ “new and weak” and “small and weak” characteristics in constructing the entrepreneurial environment. Specific work includes optimizing the administrative approval procedures for startups, improving the efficiency of administrative approvals, increasing tax incentives and subsidies for startups, and establishing a multi-level venture capital market. In addition, it is necessary to create a cultural atmosphere of “tolerance of failure” and promote “entrepreneurship-friendly” rules and regulations.

Second, because entrepreneurial passion plays a mediating role between the entrepreneurial institutional environment and entrepreneurial orientation, the entrepreneur should stimulate and maintain entrepreneurial passion. According to Campos (2017), entrepreneurial passion positively affects entrepreneurial activities as a lasting and positive emotion. The study results indicate that entrepreneurial passion can be an important driving force for maintaining enthusiasm and enhancing self-confidence in response to the stimulation of the entrepreneurial institutional environment. Therefore, entrepreneurs are supposed to participate actively in entrepreneurial activities. These social activities will continue to generate positive emotional evaluations, further encouraging entrepreneurs to persevere in entrepreneurial activities and form a virtuous circle. In addition, they must cultivate their entrepreneurial interests, including creating and expanding the appeal of enterprises optimizing products and services. At last, they should actively participate in entrepreneurial education and training, master entrepreneurial-related knowledge and skills, and consciously cultivate innovative, adventurous, and proactive entrepreneurial orientation.

### Shortcomings and Prospects

The primary limitations of this study are as follows. First, the study samples are obtained from the eastern entrepreneurial region of Mainland China. Although the study conclusions are supported, whether these research results apply to other areas requires further testing and support. Second, Krueger (1993) believes that external environmental cues trigger entrepreneurs’ internal cognitive assessment of the feasibility and desirability of action choices (Krueger, 1993). Entrepreneurs with regulatory orientation will also have different evaluations of their actions. Therefore, the follow-up can consider introducing entrepreneurs’ personal characteristic factors for exploration. Finally, this study focuses on entrepreneurial enterprises in the same region, and the entrepreneurial institutional environment impacts their entrepreneurial orientation. Future studies should collect extensive data samples from various industries and discuss in-depth “Entrepreneurial institutional environment is more conducive to entrepreneurship in which industries” and other issues.

## Data Availability Statement

The raw data supporting the conclusions of this article will be made available by the authors, without undue reservation.

## Author Contributions

XZ and LZ: conceptualization. XZ: methodology, software, resources, writing and original draft preparation, visualization, supervision, project administration, and funding acquisition. LZ: validation, formal analysis, investigation, and data curation. LZ and XS: writing, reviewing, and editing the manuscript. All authors have read and agreed to the published version of the manuscript.

## Conflict of Interest

The authors declare that the research was conducted in the absence of any commercial or financial relationships that could be construed as a potential conflict of interest.

## Publisher’s Note

All claims expressed in this article are solely those of the authors and do not necessarily represent those of their affiliated organizations, or those of the publisher, the editors and the reviewers. Any product that may be evaluated in this article, or claim that may be made by its manufacturer, is not guaranteed or endorsed by the publisher.
